# The Association between Sleep Problems, Sleep Medication Use, and Falls in Community-Dwelling Older Adults: Results from the Health and Retirement Study 2010

**DOI:** 10.1155/2016/3685789

**Published:** 2016-07-12

**Authors:** Yaena Min, Pramit A. Nadpara, Patricia W. Slattum

**Affiliations:** Department of Pharmacotherapy and Outcomes Science, School of Pharmacy, Virginia Commonwealth University, Richmond, VA 23298-0533, USA

## Abstract

*Background*. Very few studies have assessed the impact of poor sleep and sleep medication use on the risk of falls among community-dwelling older adults. The objective of this study was to evaluate the association between sleep problems, sleep medication use, and falls in community-dwelling older adults.* Methods*. The study population comprised a nationally representative sample of noninstitutionalized older adults participating in the 2010 Health and Retirement Study. Proportion of adults reporting sleep problems, sleep medication use, and fall was calculated. Multiple logistic regression models were constructed to examine the impact of sleep problems and sleep medication use on the risk of falls after controlling for covariates.* Results*. Among 9,843 community-dwelling older adults, 35.8% had reported a fall and 40.8% had reported sleep problems in the past two years. Sleep medication use was reported by 20.9% of the participants. Older adults who do have sleep problems and take sleep medications had a significant high risk of falls, compared to older adults who do not have sleep problems and do not take sleep medications. The other two groups also had significantly greater risk for falls.* Conclusion*. Sleep problems added to sleep medication use increase the risk of falls. Further prospective studies are needed to confirm these observed findings.

## 1. Introduction

One-third of community-dwelling adults aged 65 years and older experience a fall per year [1]. Falls can cause both fatal and nonfatal injuries. It is also associated with reduced functioning and earlier admission to nursing homes [2, 3]. In 2010, approximately 21,700 of older adults died from unintentional fall injuries [4].

One potential risk factor for falls that needs further investigation is self-reported sleep problems [5]. Sleep complaints are common in older adults, as 50% of community-dwelling older adults report poor sleep [6, 7], and studies have shown that sleep problems increase the risk of falls in older adults [5, 8–10]. Additionally, the consumption of medications to aid sleep, like sedative hypnotics, is common in older adults [11], and studies have shown such medication use to be associated with risk of falls [11, 12]. A meta-analysis found that sedatives and hypnotics (OR = 1.47; 95% CI = 1.35–1.62), antidepressants (OR = 1.68; 95% CI = 1.47–1.91), and benzodiazepines (OR = 1.57; 95% CI = 1.43–1.72) have a significant association with falls in older adults [12]. In another study with community-dwelling older adults, use of anxiolytics and sedatives hypnotics significantly increased the risk of falls in men (OR = 1.43; 95% CI = 1.22–1.67) and in women (OR = 1.33; 95% CI = 1.22–1.46), when they used the drug up to 85 days before the fall [13]. However, these studies did not consider reported sleep problems as a potential confounder when assessing the relationship.

One study has assessed the association between insomnia and hypnotics use on risk of falls among institutionalized population [14]. This study found that older adults with insomnia but who did not take hypnotics as well as those who have insomnia and who did use hypnotics had a greater risk of falls compared to those who did not have insomnia and did not use hypnotics [14]; thus it remains to be seen if such an association exists among community-dwelling older adults. There is clearly a lack of literature on whether the underlying sleep problems cause falls in older adults who take medications for sleep. Therefore, we conducted a study to evaluate if sleep problems and sleep medication use were associated with risk of falls, injurious falls, and recurrent falls in community-dwelling adults aged 65 years or older. Identifying whether sleep problems, sleep medication use, or both increase the risk of falls may further help healthcare decision-makers to better treat older adults with poor sleep and who are already at risk of falling.

## 2. Methods

### 2.1. Data Source and Study Sample

This study was a cross-sectional analysis of the 2010 Health and Retirement Study (HRS) dataset. Health and Retirement Study (HRS) is a longitudinal panel study that surveys more than 22,000 Americans every two years and it is conducted by the University of Michigan [15]. The most recent wave of 2010 data at the time was used in the study. In this study, we included all community-dwelling adults aged 65 years or older from the HRS dataset. We excluded those respondents who (1) resided in nursing homes during year 2010, (2) did not respond in the year 2010 survey, or (3) had missing values on any of the variables.

### 2.2. Outcome Variable

Falls were assessed in the HRS by asking the question, “Have you fallen down in the last two years?” The response to this question was categorized as “yes” or “no” and was used to define the outcome variable in this study. If the participants responded “yes,” they were further asked the number of times they had fallen and whether they had been injured seriously enough to need medical treatment in any of the falls.

### 2.3. Predictor Variables

In the HRS, respondents were asked about their sleep problems through four questions: (1) “How often do you have trouble falling asleep?” (2) “How often do you have trouble with waking up during the night?” (3) “How often do you have trouble with waking up too early and not being able to fall asleep again?” (4) “How often do you feel really rested when you wake up in the morning?” The responses were categorized as “most of the time,” “sometimes,” and “rarely or never.” Since the questions about sleep are asked as part of a questionnaire about their current health status, the responses reflect their current sleep issues. For this study, participants were defined as having a sleep problem, if the respondents answered “most of the time” in any of the first three questions or “rarely or never” on the fourth question [5, 16]. This approach was appropriate since the questions and responses were mostly the same with the past literature.

Sleep medication use by respondents was assessed in HRS with the question: “In the past two weeks, have you taken any medications or used other treatments to help you sleep?” The responses were categorized as “yes” or “no” and were used to identify respondents that used sleep medications in this study.

### 2.4. Covariates

Based on previous studies, the covariates that are recognized as a risk factor for falls were included in this study. Demographic variables of interest in this analysis were age, sex, race, education level, and marital status. Other variables of interest included smoking status, alcohol use, health status, number of comorbidities, limitation in activities of daily living (ADL), limitation in instrumental activities of daily living (IADL), limitation in mobility, self-rated eyesight, and presence of incontinence [17–19]. Age was categorized as “65–74 years,” “75–84 years,” and “85 years or older.” Sex was categorized as “male” and “female,” race as “Caucasian” and “others,” and marital status as “married” and “others.” Education level was estimated by the years of education and categorized as “no education,” “less than high school,” “high school,” and “more than high school.”

Smoking status was categorized as “current,” “former,” and “nonsmokers.” Use of alcohol was classified as “drinker” and “nondrinker.” Health status was self-reported and was categorized as “excellent/very good/good,” “fair,” and “poor.” Based on previous studies using HRS data, the number of comorbidities was measured by the sum of the following self-reported conditions: high blood pressure, diabetes, lung disease, heart disease, stroke, emotional or psychiatric problems, arthritis, and Alzheimer's disease [20–22]. Comorbidities were then categorized as “none,” “1-2,” “3-4,” or “5 or more.”

Information about functional status was also taken into account because it is highly related to risk of falls [17, 23]. Activities of daily living (ADL) included bathing, dressing, toileting, eating, and transferring. The number of limitations in activities of daily living (ADL) was the sum of any difficulties on these five tasks. Instrumental activities of daily living (IADL) included ability to use the phone, shopping, preparing for food, responsibility for own medications, and ability to handle finances. The number of limitations in instrumental activities of daily living (IADL) was the sum of any difficulties on these five tasks [21, 24]. Limitations in mobility included difficulty in any of the following tasks such as walking one block, walking several blocks, waking across a room, climbing one flight of stairs, and climbing several flights of stairs. Each criterion of functional limitation was categorized as “0,” “1-2,” or “3–5.” Self-rated eyesight was included because impaired vision has been reported to be a risk factor for falls [17, 23]. It was categorized as “excellent/very good/good/fair” and “poor/legally blind.” The presence of incontinence, categorized as “yes” or “no,” was also included [18].

### 2.5. Statistical Analysis

Using individual sampling weights, weighted descriptive statistics were generated and used to summarize the characteristics of the participants. Percentages were used to describe categorical variables, and mean and standard deviation (SD) were used to describe continuous variables. Chi-square analysis was conducted to determine the association among categorical variables of interest.

The effect of sleep problems and sleep medication use and their combined effect on falls were assessed by multiple logistic regression models. Multinominal logistic regression models were used to assess the association of recurrent falls or injurious falls with predictor variables with nonfallers as the reference group. The unadjusted relationship was calculated followed by adjusted relationship with all the covariates in the model. Collinearity among the covariates was assessed. In addition, the effect of each sleep problem (“trouble falling asleep,” “trouble with waking up during the night,” “trouble with waking up too early and not being able to fall asleep again,” and “trouble feeling rested when waking up in the morning”) on the risk of falls was assessed. Results were reported as odds ratios (OR) and 95% confidence intervals (CI). *p* value < 0.05 was considered significant. All statistical analyses were performed using SAS 9.4 statistical software (SAS Institute Inc., Cary, NC, USA).

## 3. Results

### 3.1. Population Characteristics

Based on our inclusion and exclusion criteria, we identified 10,354 older adults from the HRS 2010 dataset. Data from 511 respondents were excluded due to missing data, which is less than 5% of the respondents. Thus, the final dataset consisted of 9,843 participants.

Overall, 35.8% of the older adults reported a fall at least once in the last two years. Among those reporting a fall, 29.9% had serious injuries requiring medical treatment and 60.4% fell more than once. The proportion of older adults reporting sleep problems and sleep medication use was 40.8% and 20.9%, respectively.

Table 1 shows the characteristics of the participants by fallers and nonfallers. The proportion of participants with falls was higher among those aged 85 years or older and among females, compared to nonfallers. Similar results were seen in participants who self-reported their health as poor, had 5 or more comorbidities, had more activities of daily living (ADL), had instrumental activities of daily living (IADL) and mobility limitations, and self-reported their eyesight as poor/legally blind.

### 3.2. Associations between Sleep Problems, Sleep Medications, and Falls

Among individuals reporting falls, 43.3% (*N* = 1,515) reported no sleep problems and no use of sleep medications, 31.9% (*N* = 1,144) reported sleep problems but no use of sleep medications, 9.6% (*N* = 334) reported no sleep problems but still used sleep medications, and 15.2% (554) reported sleep problems and used sleep medications (Table 2).

Table 3 reports the unadjusted and adjusted associations between sleep problems, sleep medication use, and falls. Compared to participants reporting no sleep problems and no sleep medication use, those reporting sleep problems and sleep medication use had significantly increased risk of falls (OR = 1.79; 95% CI = 1.54–2.08). However, after adjusting for covariates, older adults with no sleep problems and sleep medication use had the highest increase in the risk of falls compared to the other groups. All of the associations were attenuated after adjusting for the covariates in the full model but remained significant.

### 3.3. Associations between Sleep Problems, Sleep Medications, and Injurious Falls

The falls were further categorized into injurious falls and noninjurious falls. Among the total sample, 10.7% experienced injurious falls while 25.1% experienced noninjurious falls. Greater proportion of injurious fallers (49.0%, *N* = 520) reported sleep problems than noninjurious fallers (46.4%, *N* = 1,178) and nonfallers (37.2%, *N* = 2,362). Similarly, greater proportion of injurious fallers (30.0%, *N* = 309) reported using sleep medications than noninjurious fallers (22.6%, *N* = 579) and nonfallers (18.8%, *N* = 1,140). Compared with nonusers, sleep medication users had a 40% higher risk of having injurious falls. All the groups were more likely to experience injurious falls compared with the group with no sleep problems and no use of sleep medications. However, the effect of sleep problems and sleep medication use did not increase the risk of noninjurious falls.  Table 4 reports the relationship between sleep problems, sleep medication use, and injurious falls.

### 3.4. Associations between Sleep Problems, Sleep Medications, and Recurrent Falls

Recurrent falls were defined as falling more than once in the past two years. Among the total sample, 14.2% were single fallers, and 21.7% were recurrent fallers. Participants using medications or treatments for sleep were approximately 18.8% (*N* = 1,140) among nonfallers, 21.6% (*N* = 305) among single fallers, and 26.9% (*N* = 583) in recurrent fallers. Recurrent fallers had higher proportion of those with sleep problems (51.8%, *N* = 1,129) compared with single fallers (40.2%, *N* = 569) and nonfallers (37.2%, *N* = 2,362). After combining sleep problems and sleep medication use, recurrent fallers had a higher proportion of those with both sleep problems and sleep medication use (17.7%, *N* = 388) compared with single fallers (11.5%, *N* = 166) and nonfallers (10.8%, *N* = 649). Compared with participants with no sleep problems, those with sleep problems were 26% more likely to have recurrent falls. Similarly, compared with nonusers of sleep medications, users had 21% higher odds of having recurrent falls. However, sleep problems and sleep medication use did not affect the risk of nonrecurrent falls. In addition, the group with sleep problems and sleep medication use had a significantly higher risk of recurrent falls compared with the group with no sleep problems and no sleep medication use.  Table 5 reports the relationship between sleep problems, sleep medication use, and recurrent falls.

### 3.5. Association between Type of Reported Sleep Problem and Falls

Among individuals reporting a fall, 16.9% (*N* = 624) reported having trouble falling asleep, 31.1% (*N* = 1,089) reported trouble waking up during the night, 15.6% (*N* = 565) reported trouble with waking up too early and not being able to fall asleep again, and 14.8% (*N* = 530) reported trouble feeling rested when waking up in the morning.  Table 6 reports the relationship between type of sleep problem and the risk for falls. The risk of falls was higher among respondents reporting any of the four sleep problems in the unadjusted associations. However, after adjusting for covariates including sleep medication use, only respondents with “trouble falling asleep” had an increased risk of falls.

## 4. Discussion and Conclusion

The purpose of this study was to assess the effect of sleep problems and sleep medication use and their combined effect on the risk of falls, injurious falls, and recurrent falls in community-dwelling older adults. In a representative sample of community-dwelling older adults in 2010, this study showed that 35.8% of the participants had a fall at least once in the last two years. Similar percentage of older adults had fallen compared to the prior studies which reported that one in three community-dwelling older adults has a fall per year [25]. However, what constitutes a fall was not defined further in the survey. About 5–10% of falls in community-dwelling older adults result in severe head injury and joint dislocation and additional 5% in a fracture [26]. Approximately 30% of falls led to serious injuries in this study; thus it is possible that respondents were more likely to remember to report falls that required medical treatments. Additionally, this study found that 40.8% report sleep problems. The prevalence was similar compared to previous studies [6, 7]. Only 20.9% used medications or other treatments to help sleep in the past two weeks and among those who have sleep problems as we have defined in this study, only 30.4% used sleep medications. Many older adults may continue to take sleep medications despite continued sleep problems. No significant differences in sleep quality between older adults who take benzodiazepine-receptor agonists and those who take placebos have been reported [11], and there is lack of evidence of significant treatment effect of using sedative hypnotics on a long-term basis [27].

Older adults with reported sleep problems had a significant increase in the risk of falls after adjusting for covariates (OR = 1.13; 95% CI = 1.01–1.25). The possible mechanisms could be that poor sleep may cause daytime sleepiness, cognitive dysfunction, and slower response time which could result in falls [5, 14]. Although the risk attenuated after considering covariates, the risk was still observed. However, older adults who used sleep medications or other treatments for sleep were not at increased risk of falls after adjusting for covariates. A study by Ensrud and colleagues which followed community-dwelling older women on hypnotics for one year for incidence of falls found that women taking benzodiazepines (multivariate odds ratio, MOR = 1.34; 95% CI = 1.09–1.63), antidepressants (MOR = 1.54; 95% CI = 1.14–2.07), and antiepileptics (MOR = 2.56; 95% CI = 1.49–4.41) were more likely to experience falls at least once [28]. Sedative hypnotics could increase falls by the effect on postural instability, psychomotor impairment, and cognitive dysfunction [29]. Furthermore, the risk of falls associated with these medications could be increased because of age-related changes in physiology resulting in altered pharmacokinetics and pharmacodynamics in older adults [29]. Therefore, the lack of significance in the finding could again be due to a short time frame of documented sleep medication use relative to the time frame for reported falls and lack of definition of sleep medication and treatments for the respondents in the survey.

Additionally, there was increased risk of falls among older adults who had sleep problems and who used sleep medications. However, after adjusting for covariates, older adults with no sleep problems and who used sleep medications had the highest risk of falls compared to other groups. This finding suggests that sleep medications did not have a protective effect on falls when considering sleep problems in the association. A study by Avidan and colleagues assessed the risk of falls in the same four groups with cross-tabulation of insomnia and use of sedative hypnotics: (1) insomnia, hypnotic use, (2) insomnia, no hypnotic use, (3) no insomnia, hypnotic use, and (4) no insomnia, no hypnotic use [14]. However, the results are inconsistent with the current study. Avidan and colleagues found that insomniacs who did not take hypnotics (OR = 1.55; 95% CI = 1.41–1.71) and insomniacs who took hypnotics had a significant increase in the risk of falls (OR = 1.32; 95% CI = 1.02–1.70) [14]. Insomnia without hypnotics had the highest risk of falls compared to any other groups and hypnotic use had a protective effect on falls in older adults, suggesting that the condition of insomnia might have been better treated with the medication resulting in fewer falls [14]. Although the study by Avidan and colleagues and the current study have conflicting results, the presence of sleep problems such as insomnia was found as a key risk factor for falls.

The risk of injurious falls associated with sleep problems and sleep medication use was also assessed with a multinominal logistic regression model. The effect of sleep problems and sleep medication use on the risk of injurious falls showed that the group with no sleep problems and sleep medication use had the highest risk of injurious falls compared to those with sleep problems and no sleep medication use and to those with sleep problems and sleep medication use compared to the reference group. This pattern was similar to the risk of falls generally. A prospective cohort study also found a significant increased risk of injurious falls related to the use of sedative or hypnotics (RR = 1.50; 95% CI = 1.03–2.19) [30], but other studies did not find sleep problems to be related to falls that required medical attention for an injury [31, 32]. Moreover, the risk of recurrent falls was studied. The risk of recurrent falls was seen in older adults with sleep problems and no sleep medication use and in those with sleep problems and sleep medication use but it was not seen in those with no sleep problems and sleep medication use. This could be due to the low sample size in that group. None of sleep problems and sleep medication use was associated with risk of single fall and this could be related to differences in physical performance [33] and risk factors of falls [34] between single fallers and recurrent fallers.

In this study, the risk of falls by type of reported sleep problem among older adults was also assessed. Each sleep problem had a significant increase in the unadjusted risk of falls; however, only “trouble falling asleep” significantly increased the risk of falls after adjusting for covariates including the use of sleep medications. We do not know when falls occurred in these older adults, but since they had trouble falling asleep even though they took medications or treatment for sleep, we can assume that they could have walked around during the night in a hyperarousal state which can increase the risk of falls. A study by Brassington and colleagues evaluated the same aspects of sleep disturbance in older adults as in the current study except for one type of sleep problem, which was “trouble feeling rested when waking up in the morning,” and found that difficulty in falling asleep at night (OR = 1.53; 95% CI 1.04–2.24), waking up during the night (OR = 1.91; 95% CI = 1.44–2.54), and waking up too early in the morning and not being able to fall asleep again (OR = 1.64; 95% CI = 1.11–2.42) were significantly associated with the occurrence of falls after adjusting for covariates including a use of prescription medication [5]. The number of covariates that were adjusted for was fewer than in the current study and therefore may not have adjusted for the effect of confounders such as ADLs, IADLs, and mobility. In contrast, Teo and colleagues evaluated the same categories of nighttime sleep problems and used the same scale as Brassington and colleagues and reported conflicting findings [16]. Teo and colleagues did not find a significant increase in the risk of falls in the same nighttime sleep problems after adjusting for the covariates [16]. While results from our study add to the finding from previous studies, further prospective research in community-dwelling older adults is needed to accurately quantify the risk of falls by type of sleep problem.

Some strengths of this study may be noted. Since the HRS study had representative sample of older adults, national estimates were obtained. Also, several risk factors such as ADL and IADL were controlled as covariates in this study. However, as with any study, this study has limitations. First, since information was self-reported, there could be misclassification bias or recall bias. Second, because this is a cross-sectional study based on self-reported information, causal relationships cannot be explored in this study. Third, falls and medications or other treatments to help sleep were not clearly defined in the HRS survey. Variations in the definition of falls could result in misclassification bias. Fourth, unlike other questions where respondents were asked to provide information on their current sleep issues and sleep medication use, the questions on assessing falls were asked based on their experience during the last two years. The differences in the time interval may result in lack of temporal relationship between drug exposure and the outcome. Still the study results add valuable information to the current literature by shedding light on sleep problem, sleep medication use, and falls in community-dwelling older adults. Finally, information on the circumstances of falls was not collected in HRS but this information would have been useful and should be collected in future studies.

In conclusion, this study found that sleep problems, use of sleep medications, and occurrence of falls are common among community-dwelling older adults. This study adds to the literature by assessing the combined effect of sleep problems and sleep medication use on risk of falls, injurious falls, and recurrent falls in community-dwelling older adults by grouping the participants. Older adults without sleep problems and taking sleep medication had the highest risk of falls compared to other groups of those with sleep problems and taking sleep medications, those with sleep problems and not taking sleep medications, and those without sleep problems and not taking sleep medications. Healthcare professionals should consider medication-associated risk when treating sleep problems in community-dwelling older adults. Moreover, more studies are needed to examine this association in community-dwelling older adults.

## Figures and Tables

**Table 1:** Characteristics of community-dwelling older adults by reported sleep problems and sleep medication use and falls in the US, 2010.

Variable	Total *N* (%)	Fallers^a^				Nonfallers			
		Sleep problems^b^, %		Sleep medication use^c^, %		Sleep problems, %		Sleep medication use, %	
		Yes	No	Yes	No	Yes	No	Yes	No
Total	9843	1698	1849	888	2659	2362	3934	1140	5156

Age (years)^†^									
65–74	5112 (55.5)	47.8	51.1	47.5	50.2	55.8	60.7	56.4	59.4
75–84	3505 (32.3)	36.5	33.5	36.7	34.3	33.1	29.5	32.5	30.5
>85	1226 (12.2)	15.7	15.4	15.8	15.5	11.1	9.8	11.1	10.1

Sex^*∗*^									
Male	4195 (43.7)	38.3	42.7	30.9	43.9	42.9	46.9	35.8	47.6
Female	5648 (56.3)	61.7	57.3	69.1	56.1	57.1	53.1	64.2	52.4

Race^†^									
Caucasian	8207 (89.9)	91.8	91.7	94.8	90.8	89.9	88.2	94.1	87.6
Others	1636 (10.1)	8.2	8.3	5.2	9.2	10.1	11.8	5.9	12.4

Marital status^†^									
Married	5594 (54.9)	48.2	51.9	51.1	49.8	54.3	59.5	56.0	57.9
Others	4249 (45.1)	51.8	48.1	48.9	50.2	45.7	40.5	44.0	42.1

Education									
No education	66 (0.4)	0.5	0.3	0.4	0.4	0.7	0.2	0.2	0.5
<High school	2329 (19.9)	24.5	18.4	21.4	21.2	21.6	17.7	16.5	19.8
High school	3448 (35.4)	32.6	38.2	34.8	35.8	35.3	35.3	34.0	35.5
>High school	4000 (44.3)	42.4	43.1	43.4	42.6	42.5	46.8	49.3	44.2

Smoking									
Current smoker	846 (9.4)	9.8	8.2	8.3	9.2	10.4	9.2	8.0	10.1
Former smoker	4769 (48.4)	49.9	48.6	50.7	48.7	49.7	46.8	46.8	48.1
Nonsmoker	4228 (42.2)	40.3	43.2	41.0	42.1	39.9	44.0	45.2	41.8

Alcohol^†^									
Current drinker	4698 (50.8)	45.6	48.5	43.4	48.4	50.3	54.4	53.3	52.8
Nondrinker	5145 (49.2)	54.4	51.5	56.6	51.6	49.7	45.6	46.7	47.2

Self-reported health^†^									
E/VG/G	7076 (74.5)	54.7	72.9	55.8	67.1	71.4	85.2	74.9	81.3
Fair	2022 (18.6)	28.6	20.7	26.8	23.7	21.3	11.9	18.1	14.8
Bad	745 (6.9)	16.7	6.4	17.4	9.2	7.3	2.8	7.0	3.9

Number of comorbidities^†^									
0	661 (7.6)	2.8	5.4	2.6	4.7	5.9	11.7	4.6	10.7
1-2	4730 (49.4)	36.3	47.8	35.2	44.7	48.7	56.1	51.7	53.7
3-4	3753 (36.2)	46.5	38.5	47.7	40.5	39.1	29.1	38.8	31.4
5 or more	699 (6.8)	14.4	8.3	14.5	10.1	6.3	3.1	4.9	4.2

ADL limitations^†^									
None	7964 (82.4)	64.2	77.5	63.8	73.6	84.0	91.5	87.0	89.1
1-2	1330 (12.6)	24.1	16.4	23.9	18.8	11.4	6.6	8.6	8.3
3–5	549 (5.0)	11.7	6.1	12.3	7.6	4.6	1.9	4.4	2.6

IADL limitations^†^									
None	8070 (83.4)	69.1	78.7	68.5	76.0	85.3	90.6	84.8	89.5
1-2	1254 (12.1)	21.4	15.3	21.8	17.0	11.7	7.0	11.7	8.0
3–5	519 (4.5)	9.5	6.0	9.7	7.0	3.0	2.4	3.5	2.5

Mobility limitations^†^									
None	4137 (44.3)	24.7	39.5	26.1	34.6	40.8	56.8	40.7	53.2
1-2	3485 (34.6)	35.2	35.8	34.0	36.0	38.2	31.7	39.9	32.8
3–5	2221 (21.1)	40.1	24.7	39.9	29.4	21.0	11.5	19.4	14.0

Self-reported eyesight^†^									
E/VG/G/fair	9161 (93.8)	87.6	92.8	88.1	91.1	94.2	96.6	94.6	95.9
Bad/legally blind	682 (6.2)	12.4	7.2	11.9	8.9	5.8	3.4	5.4	4.1

Incontinence^†^									
Yes	2806 (28.6)	45.3	30.0	43.1	35.3	31.2	19.4	33.4	21.6
No	7037 (71.4)	54.7	70.0	56.9	64.7	68.8	80.6	66.6	78.4

*Note*. Data source: 2010 HRS (RAND HRS data file and 2010 HRS core physical health file).

Results are reported in weighted percentages.

^*∗*^Chi-test *p* value < 0.05; ^†^< 0.0001.

^a^Fallers = participants who reported having a fall at least once in the previous two years.

^b^Sleep problems = participants who have trouble “most of the time” in any of sleep problems: “trouble falling asleep,” “trouble with waking up during the night,” and “trouble with waking up too early and not being able to fall asleep again,” or “rarely or never” on sleep problem: “trouble feeling rested when waking up in the morning.”

^c^Sleep medication use = participants who have reported taking any medications or using other treatments to help sleep.

ADL = activities of daily living; IADL = instrumental activities of daily living; E/VG/G = excellent/very good/good.

**Table 2:** Distribution of predictor variables against falls in community-dwelling older adults in the US, 2010.

Variables	Fallers	Nonfallers
	*N* (%)	*N* (%)
Sleep problems		
Yes	1698 (47.2)	2362 (37.2)
No	1849 (52.8)	3934 (62.8)

Sleep medication use		
Yes	888 (24.8)	1140 (18.8)
No	2659 (75.2)	5156 (81.2)

Sleep problems + sleep medication use		
No sleep problem + no sleep med use^a^	1515 (43.3)	3443 (54.8)
No sleep problem + sleep med use^b^	334 (9.6)	491 (8.0)
Sleep problem + no sleep med use^c^	1144 (31.9)	1713 (26.4)
Sleep problem + sleep med use^d^	554 (15.2)	649 (10.8)

*Note*. Data source: 2010 HRS dataset (RAND HRS data file and 2010 HRS core physical health file). Results are reported in weighted percentages.

Column percentages are significantly different by Rao-Scott chi-square test *p* < 0.05 and cannot be calculated based on raw numbers.

^a^No sleep problem + no sleep med use = participants who reported no sleep problems and no use of sleep medications.

^b^No sleep problem + sleep med use = participants who reported no sleep problems but use of sleep medications.

^c^Sleep problem + no sleep med use = participants who reported sleep problems and no use of sleep medications.

^d^Sleep problem + sleep med use = participants who reported sleep problems and use of sleep medications.

**Table 3:** Association between sleep problems, sleep medication use, and falls in community-dwelling older adults in the US, 2010.

Variables	Unadjusted odds ratios (95% CI)	Adjusted odds ratios^a^ (95% CI)
Sleep problems		
Yes	1.51^‡^ (1.37–1.65)	1.13^*∗*^ (1.01–1.25)
No	Ref.	Ref.

Sleep medication use		
Yes	1.43^‡^ (1.26–1.62)	1.14 (1.00–1.31)
No	Ref.	Ref.

Sleep problems + sleep medication use		
No sleep problem + no sleep med use^b^	Ref.	Ref.
No sleep problem + sleep med use^c^	1.52^‡^ (1.31–1.78)	1.24^†^ (1.05–1.47)
Sleep problem + no sleep med use^d^	1.53^‡^ (1.36–1.73)	1.16^*∗*^ (1.02–1.32)
Sleep problem + sleep med use^e^	1.79^‡^ (1.54–2.08)	1.19^*∗*^ (1.01–1.41)

*Note*. Data source: 2010 HRS dataset (RAND HRS data file and 2010 HRS core physical health file).

^*∗*^ *p* value < 0.05, ^†^< 0.01, and ^‡^< 0.0001.

Ref. = reference.

^a^Full model: adjusted for age, sex, education, marital status, race, self-reported health, alcohol, number of comorbidities, limitations in instrumental activities of daily living (IADL), limitations in activities of daily living (ADL), limitations in mobility, self-rated eyesight, and incontinence.

^b^No sleep problem + no sleep med use = participants who reported no sleep problems and no use of sleep medications.

^c^No sleep problem + sleep med use = participants who reported no sleep problems but use of sleep medications.

^d^Sleep problem + no sleep med use = participants who reported sleep problems and no use of sleep medications.

^e^Sleep problem + sleep med use = participants who reported sleep problems and use of sleep medications.

**Table 4:** Association between sleep problems, sleep medication use, and injurious falls in community-dwelling older adults in the US, 2010.

Variables	Noninjurious fallers	Injurious fallers
	Adjusted OR (95% CI)	Adjusted OR^e^ (95% CI)
Sleep problems		
Yes	1.12 (1.00–1.25)	1.16 (0.98–1.36)
No	Ref.	Ref.

Sleep medication use		
Yes	1.04 (0.89–1.22)	1.40^†^ (1.15–1.71)
No	Ref.	Ref.

Sleep problems + sleep medication use		
No sleep problem + no sleep med use^a^	Ref.	Ref.
No sleep problem + sleep med use^b^	1.05 (0.86–1.30)	1.76^‡^ (1.40–2.20)
Sleep problem + no sleep med use^c^	1.13 (0.99–1.29)	1.25^*∗*^ (1.02–1.54)
Sleep problem + sleep med use^d^	1.12 (0.92–1.35)	1.40^*∗*^ (1.08–1.82)

*Note*. Data source: 2010 HRS dataset (RAND HRS data file and 2010 HRS core physical health file).

^*∗*^ *p* value < 0.05, ^†^< 0.01, and ^‡^< 0.0001.

Ref. = reference.

^a^No sleep problem + no sleep med use = participants who reported no sleep problems and no use of sleep medications.

^b^No sleep problem + sleep med use = participants who reported no sleep problems but use of sleep medications.

^c^Sleep problem + no sleep med use = participants who reported sleep problems and no use of sleep medications.

^d^Sleep problem + sleep med use = participants who reported sleep problems and use of sleep medications.

^e^Full model: adjusted for age, gender, education, marital status, race, self-reported health, alcohol, number of comorbidities, limitations in IADL, limitations in ADL, limitations in mobility, self-rated eyesight, and incontinence.

**Table 5:** Association between sleep problems, sleep medication use, and recurrent falls in community-dwelling older adults in the US, 2010.

Variables	Single fallers	Recurrent fallers
	Adjusted OR (95% CI)	Adjusted OR^e^ (95% CI)
Sleep problems		
Yes	0.97 (0.83–1.12)	1.26^†^ (1.12–1.42)
No	Ref.	Ref.

Sleep medication use		
Yes	1.05 (0.89–1.23)	1.21^*∗*^ (1.04–1.42)
No	Ref.	Ref.

Sleep problems + sleep medication use		
No sleep problem + no sleep med use^a^	Ref.	Ref.
No sleep problem + sleep med use^b^	1.24 (0.99–1.55)	1.25 (1.00–1.58)
Sleep problem + no sleep med use^c^	1.03 (0.86–1.23)	1.27^†^ (1.10–1.47)
Sleep problem + sleep med use^d^	0.93 (0.75–1.16)	1.40^†^ (1.17–1.68)

Data source: 2010 HRS dataset (RAND HRS data file and 2010 HRS core physical health file).

^*∗*^ *p* value < 0.05, ^†^< 0.01.

Ref. = reference.

^a^No sleep problem + no sleep med use = participants who reported no sleep problems and no use of sleep medications.

^b^No sleep problem + sleep med use = participants who reported no sleep problems but use of sleep medications.

^c^Sleep problem + no sleep med use = participants who reported sleep problems and no use of sleep medications.

^d^Sleep problem + sleep med use = participants who reported sleep problems and use of sleep medications.

^e^Full model: adjusted for age, gender, education, marital status, race, self-reported health, alcohol, number of comorbidities, limitations in IADL, limitations in ADL, limitations in mobility, self-rated eyesight, and incontinence.

**Table 6:** Associations between type of sleep problems and falls in community-dwelling older adults in the US, 2010.

Variables	Unadjusted odds ratios (95% CI)	Adjusted odds ratios^a^ (95% CI)
Trouble falling asleep		
Yes	1.75^‡^ (1.54–1.98)	1.22^†^ (1.07–1.40)
No	Ref.	Ref.

Trouble with waking up during the night		
Yes	1.35^‡^ (1.22–1.50)	1.07 (0.96–1.20)
No	Ref.	Ref.

Trouble with waking up too early and not being able to fall asleep again		
Yes	1.50^‡^ (1.31–1.71)	1.12 (0.98–1.30)
No	Ref.	Ref.

Trouble feeling rested when waking up in the morning		
Yes	1.54^‡^ (1.34–1.78)	0.98 (0.84–1.14)
No	Ref.	Ref.

*Note*. Data source: 2010 HRS dataset (RAND HRS data file and 2010 HRS core physical health file).

*p* value < 0.05, ^†^< 0.01, and ^‡^< 0.0001.

Ref. = reference.

^a^Full model: adjusted for age, sex, education, marital status, race, self-reported health, alcohol, number of comorbidities, limitations in instrumental activities of daily living (IADL), limitations in activities of daily living (ADL), limitations in mobility, self-rated eyesight, incontinence, and sleep medication use.

## References

[1] Stel V. S., Pluijm S. M. F., Deeg D. J. H., Smit J. H., Bouter L. M., Lips P. (2003). A classification tree for predicting recurrent falling in community-dwelling older persons. *Journal of the American Geriatrics Society*.

[2] Panel on Prevention of Falls in Older Persons (2011). Summary of the Updated American Geriatrics Society/British Geriatrics Society clinical practice guideline for prevention of falls in older persons. *Journal of the American Geriatrics Society*.

[3] Rubenstein L. Z., Josephson K. R., Robbins A. S. (1994). Falls in the nursing home. *Annals of Internal Medicin*.

[4] Centers for Disease Control and Prevention http://www.cdc.gov/injury/wisqars/.

[5] Brassington G. S., King A. C., Bliwise D. L. (2000). Sleep problems as a risk factor for falls in a sample of community-dwelling adults aged 64–99 years. *Journal of the American Geriatrics Society*.

[6] Foley D. J., Monjan A. A., Brown S. L., Simonsick E. M., Wallace R. B., Blazer D. G. (1995). Sleep complaints among elderly persons: an epidemiologic study of three communities. *Sleep*.

[7] Foley D., Ancoli-Israel S., Britz P., Walsh J. (2004). Sleep disturbances and chronic disease in older adults: results of the 2003 National Sleep Foundation Sleep in America Survey. *Journal of Psychosomatic Research*.

[8] Hill E. L., Cumming R. G., Lewis R., Carrington S., Le Couteur D. G. (2007). Sleep disturbances and falls in older people. *Journals of Gerontology—Series A Biological Sciences and Medical Sciences*.

[9] Helbig A. K., Döring A., Heier M. (2013). Association between sleep disturbances and falls among the elderly: results from the german cooperative health research in the region of augsburg-age study. *Sleep Medicine*.

[10] Stone K. L., Ancoli-Israel S., Blackwell T. (2008). Actigraphy-measured sleep characteristics and risk of falls in older women. *Archives of Internal Medicine*.

[11] Glass J., Lanctôt K. L., Herrmann N., Sproule B. A., Busto U. E. (2005). Sedative hypnotics in older people with insomnia: meta-analysis of risks and benefits. *British Medical Journal*.

[12] Woolcott J. C., Richardson K. J., Wiens M. O. (2009). Meta-analysis of the impact of 9 medication classes on falls in elderly persons. *Archives of Internal Medicine*.

[13] Modén B., Merlo J., Ohlsson H., Rosvall M. (2010). Psychotropic drugs and falling accidents among the elderly: a nested case control study in the whole population of Scania, Sweden. *Journal of Epidemiology and Community Health*.

[14] Avidan A. Y., Fries B. E., James M. L., Szafara K. L., Wright G. T., Chervin R. D. (2005). Insomnia and hypnotic use, recorded in the minimum data set, as predictors of falls and hip fractures in Michigan nursing homes. *Journal of the American Geriatrics Society*.

[15] Servais M. A. (2004). *Overview of HRS Public Data Files for Cross-Sectional and Longitudinal Analysis*.

[16] Teo J. S. H., Briffa N. K., Devine A., Dhaliwal S. S., Prince R. L. (2006). Do sleep problems or urinary incontinence predict falls in elderly women?. *Australian Journal of Physiotherapy*.

[17] Fabre J. M., Ellis R., Kosma M., Wood R. H. (2010). Falls risk factors and a compendium of falls risk screening instruments. *Journal of Geriatric Physical Therapy*.

[18] van Nieuwenhuizen R. C., van Dijk N., van Breda F. G. (2010). Assessing the prevalence of modifiable risk factors in older patients visiting an ED due to a fall using the CAREFALL Triage Instrument. *American Journal of Emergency Medicine*.

[19] Tinetti M. E., Kumar C. (2010). The patient who falls: ‘it's always a trade-off’. *Journal of the American Medical Association*.

[20] Chen T.-Y., Janke M. C. (2014). Predictors of falls among community-dwelling older adults with cancer: results from the health and retirement study. *Supportive Care in Cancer*.

[21] Gure T. R., McCammon R. J., Cigolle C. T., Koelling T. M., Blaum C. S., Langa K. M. (2012). Predictors of self-report of heart failure in a population-based survey of older adults. *Circulation: Cardiovascular Quality and Outcomes*.

[22] Kerr E. A., Heisler M., Krein S. L. (2007). Beyond comorbidity counts: how do comorbidity type and severity influence diabetes patients' treatment priorities and self-management?. *Journal of General Internal Medicine*.

[23] Guideline for the Prevention of Falls in Older Persons (2001). American Geriatrics Society, British Geriatrics Society, and American Academy of orthopaedic surgeons panel on falls prevention. *Journal of the American Geriatrics Society*.

[24] Gaines J. M., Poey J. L., Marx K. A., Parrish J. M., Resnick B. (2011). Health and medical services use: a matched case comparison between CCRC residents and national health and retirement study samples. *Journal of Gerontological Social Work*.

[25] Tromp A. M., Pluijm S. M. F., Smit J. H., Deeg D. J. H., Bouter L. M., Lips P. (2001). Fall-risk screening test: a prospective study on predictors for falls in community-dwelling elderly. *Journal of Clinical Epidemiology*.

[26] Bleijlevens M. H. C., Diederiks J. P. M., Hendriks M. R. C., Van Haastregt J. C. M., Crebolder H. F. J. M., Van Eijk J. T. (2010). Relationship between location and activity in injurious falls: An Exploratory Study. *BMC Geriatrics*.

[27] Unbehaun T., Spiegelhalder K., Hirscher V., Riemann D. (2010). Management of insomnia: update and new approaches. *Nature and Science of Sleep*.

[28] Ensrud K. E., Blackwell T. L., Mangione C. M. (2002). Central nervous system-active medications and risk for falls in older women. *Journal of the American Geriatrics Society*.

[29] Allain H., Bentué-Ferrer D., Polard E., Akwa Y., Patat A. (2005). Postural instability and consequent falls and hip fractures associated with use of hypnotics in the elderly: a comparative review. *Drugs & Aging*.

[30] Stenbacka M., Jansson B., Leifman A., Romelsjö A. (2002). Association between use of sedatives or hypnotics, alcohol consumption, or other risk factors and a single injurious fall or multiple injurious falls: A Longitudinal General Population Study. *Alcohol*.

[31] Byles J. E., Mishra G. D., Harris M. A., Nair K. (2003). The problems of sleep for older women: changes in health outcomes. *Age and Ageing*.

[32] Grundstrom A. C., Guse C. E., Layde P. M. (2012). Risk factors for falls and fall-related injuries in adults 85 years of age and older. *Archives of Gerontology and Geriatrics*.

[33] Boyé N. D. A., Mattace-Raso F. U. S., Van Lieshout E. M. M., Hartholt K. A., Van Beeck E. F., Van der Cammen T. J. M. (2015). Physical performance and quality of life in single and recurrent fallers: data from the Improving Medication Prescribing to Reduce Risk of Falls study. *Geriatrics and Gerontology International*.

[34] Wu T.-Y., Chie W.-C., Yang R.-S., Kuo K.-L., Wong W.-K., Liaw C.-K. (2013). Risk factors for single and recurrent falls: a prospective study of falls in community dwelling seniors without cognitive impairment. *Preventive Medicine*.

